# Predictors of long-term maintenance of changes in fruit and vegetables intake: a longitudinal analysis within a randomized controlled community trial

**DOI:** 10.1186/s12966-026-01875-3

**Published:** 2026-02-07

**Authors:** Maria Cecília Ramos de Carvalho, Aline Cristine Souza Lopes

**Affiliations:** 1 Grupo de Pesquisa de Intervenções Em Nutrição, Universidade Federal de Minas Gerais, 190 Alfredo Balena Avenue, Nursing School, Room 102, Santa Efigênia, Belo Horizonte, MG 30130-100 Brazil; 2 Escola de Enfermagem, Departamento de Nutrição, Grupo de Pesquisa de Intervenções Em Nutrição, Universidade Federal de Minas Gerais, 190 Alfredo Balena Avenue, Nursing School, Room 316, Santa Efigênia, Belo Horizonte, MG 30130-100 Brazil

**Keywords:** Fruit, Longitudinal Studies, Primary Health Care, Randomized Controlled Trial, Vegetables

## Abstract

**Background:**

Individual characteristics can be associated with maintaining adequate FV intake over time. Thus, we aimed to identify factors associated with maintaining changes in fruit/vegetable (FV) intake at 48 months.

**Methods:**

Longitudinal analysis of data from a randomized trial carried out in a health promotion service, including individuals with positive changes in FV intake after intervention. FV intake was assessed at 48 months, compared to 12 months to identify whether maintenance was associated with demographics, health data, stages of change, self-efficacy, and decisional balance, using multivariate regression.

**Results:**

We included 2,232 participants, 88.4% were women, 46.6% were maintainers at 48 months. Maintainers were older, had lower schooling and baseline FV intake, had been in the service for longer, and increased FV intake between 12 and 48 months while non-maintainers decreased it. Being older or in the service for 36 + months were associated with 1% and 30% higher odds of maintenance; higher baseline FV intake and self-efficacy were associated with 1% and 2% lower odds of maintenance.

**Conclusions:**

Maintenance of changes in FV intake was associated with higher age, a longer time in the service, and lower baseline FV intake and self-efficacy. Health promotion services should aim for participant retention, and interventionists should pay attention to participants who might face more barriers for maintenance.

**Trial registration:**

RBR-9h7ckx. Date of registration: August 12, 2015.

## Background

Fruit and vegetables (FV) are important for a healthy diet, due to their health promoting and disease preventing potential [1]. Nonetheless, population FV intake in Brazil and worldwide does not reach the recommended 400 g/day, which correspond to five servings [2, 3]. Adequate FV intake should be sustained in the long-term in order to improve cardiovascular health and reduce mortality risk [1]. Therefore, community-based interventions are necessary to increase FV intake among populations, and the maintenance of such increases should be assessed over time to inform health surveillance, longitudinal studies, and for planning and assessing the effectiveness of nutrition interventions on the medium- and long-term, especially in Primary Health Care (PHC) settings [4].

Age, income, and aspects of eating behavior, such as Transtheoretical Model (TTM) constructs – stages of change, self-efficacy and decisional balance – have been shown to predict long-term maintenance of positive intervention outcomes [5–9]. These predictors can be targeted to increase the success of food and nutrition education interventions [10–12].

According to the TTM, stages of change represent an individual’s readiness to change behavior, and five stages of change are well-established: pre-contemplation, contemplation, preparation, action, and maintenance. For any behavior, individuals who are not ready to initiate change within six months are classified in pre-contemplation. Those who intend to change within six months but are not confident to start within 30 days are classified in contemplation. Individuals who have plans to start changing within 30 days are classified in preparation. If an individual has started changing their behavior, they may be classified in action, if they have maintained change for up to six months, or in maintenance, if change has been maintained for over six months [13]. Stage progression is mediated by self-efficacy and decisional balance. Self-efficacy represents an individual’s confidence in their ability to perform behavior change when faced with challenges. Decisional balance represents the relative importance that an individual gives to pros and cons of changing [13].

Since individual characteristics and TTM constructs can be associated with maintaining adequate FV intake over time, longitudinal assessments are necessary to identify predictors of long-term increased FV intake within populations [10–12]. Previous work by Mendonça et al. has shown that a TTM-based intervention delivered by trained dietitians in face-to-face group sessions over seven months was associated with increases in FV intake at 12 months, especially among individuals with a lower FV intake at baseline [14]. However, Carvalho et al. have demonstrated that the intervention was not associated with maintaining these increases after 48 months of follow-up [15].

## Methods

We aimed to identify other individual factors associated with the maintenance of increases in FV intake at 48 months of follow-up.

### Study design, setting, and sampling

**Fig. 1:**
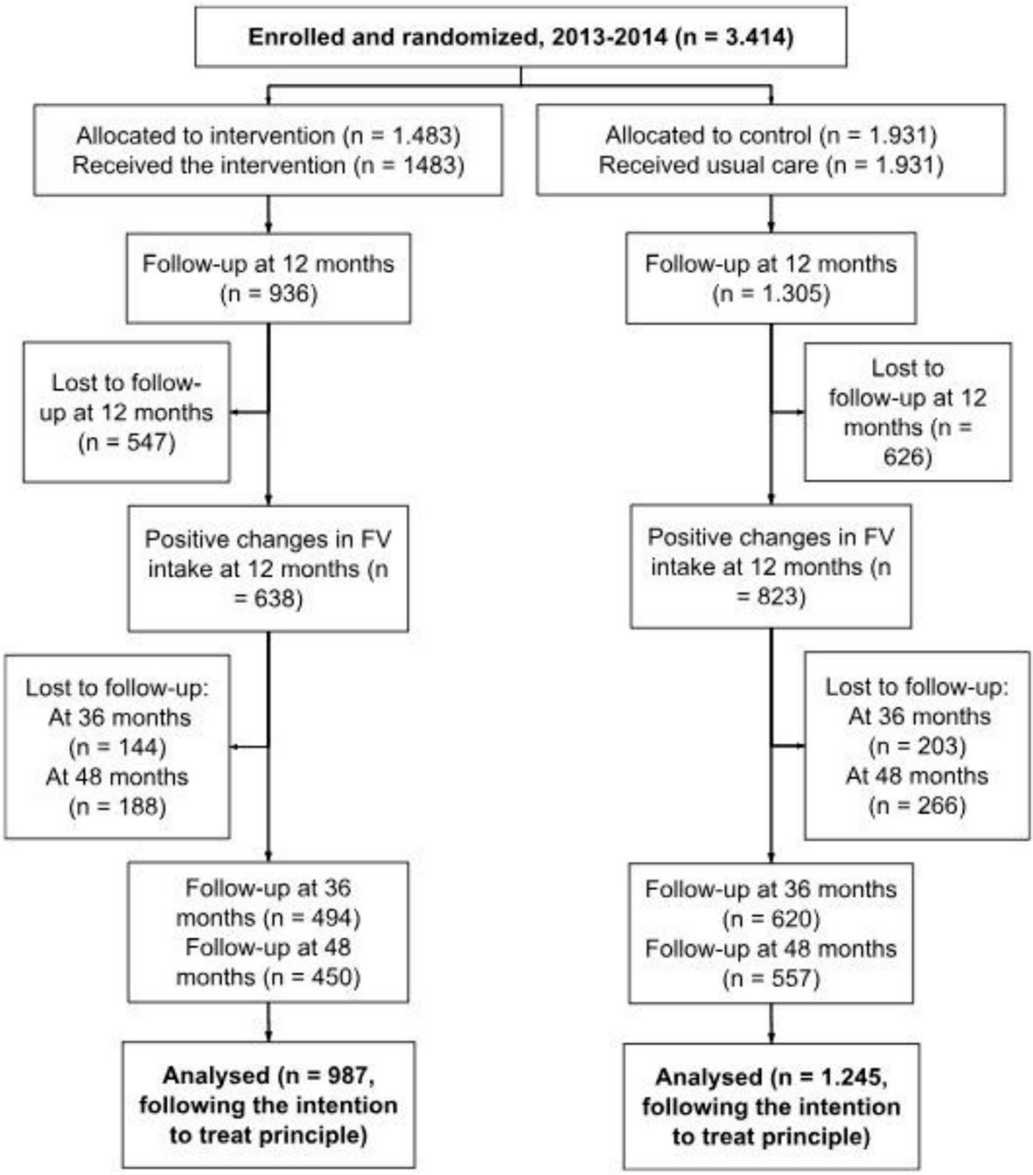
CONSORT flowchart. Belo Horizonte, Minas Gerais, Brazil, 2013–2019

The study is reported according to the Consolidated Standards of Reporting Trials (CONSORT) Statement and the Template for Intervention Description and Replication (TIDieR) Checklist (Fig. 1; Checklists). Our data come from follow-up assessments of a randomized controlled community trial (RCCT) in which we tested a group-based nutrition intervention to increase FV intake among health promotion service users. The intervention was based on TTM constructs and the pedagogy of Paulo Freire, and further information has been published by Menezes et al. [16, 17].

The RCCT was carried out in a Brazilian health promotion service called Health Academy Program *(Programa Academia da Saúde, PAS)*. PAS is part of Primary Health Care in the Unified Health System, and aims to enhance population access to physical activity and health promotion [16, 17].

The RCCT sample comprises 18 PAS units in Belo Horizonte, from which all adult or elderly participants (aged 20 years or older) and who had attended the service at least once in the previous month were eligible and invited to the trial. Individuals aged 18 or 19 were not deemed eligible due to being considered adolescents, according to the World Health Organization [18]. This sample is representative of PAS units located in medium, high, or very high vulnerability neighborhoods, with 95% confidence, and error ≤ 1.4% [16, 17].

For this study, we assessed participants who displayed some positive result in FV intake immediately after the intervention: those with a low baseline intake (< 400 g/day) who had any increase in intake at 12 months, and those with an adequate baseline intake (≥ 400 g/day) whose intake remained adequate at 12 months. Therefore, for those who had a low FV intake at baseline, intake at 12 months must be higher than at baseline for them to be included in this study. On the other hand, individuals with an adequate FV intake at baseline could be included even if their FV intake at 12 months was lower than at baseline, as long as it remained above 400 g/day.

Since we performed multiple imputation of outcome data, the number of individuals whose data were analyzed might be larger than the number of individuals who were assessed at follow-up, i.e., an individual with a positive result in FV intake at 12 months, but who was lost to follow-up at 36 or 48 months, still had their data analyzed (Fig. 1). Further details of missing data imputation methods are included in the data analysis section below.

### Data collection

Trained interviewers performed face-to-face data collection at baseline and 12 months, and computer-assisted telephone interviews at 48 months. Field supervisors worked closely with interviewers, and Data Collection Handbooks were available to ensure standardization of data collection procedures [16, 17].

We analyzed the following baseline data: sex (female/male); age (years); schooling (years); monthly *per capita* income (household income divided by the number of residents); measured height (m) and weight (kg) to calculate body mass index (BMI, kg/m^2^); self-reported hypertension and diabetes *mellitus,* attempts to lose weight (yes/no), and receipt of nutrition counseling (yes/no); FV intake (g/day); self-efficacy; decisional balance; stages of change for FV intake; time since joining HAP (difference between the date of data collection and the date each participant joined the service). Data on self-efficacy were collected at the 12 months follow-up, and short-term changes in self-efficacy were calculated as the difference between follow-up self-efficacy and baseline self-efficacy.

We estimated daily FV intake using brief questions from a national survey, which were validated with our population for face-to-face and telephone-based data collection with moderate to substantial agreement between methods [19, 20]. Instead of assessing intake of specific fruit and vegetables, the brief instrument comprises questions about overall fruit intake, and similarly, overall vegetable intake. FV were assessed as separate groups, and participants were instructed not to count fruit juices, smoothies, and starchy roots when reporting their FV intake, due to these foods’ different nutrient composition and health effects [19].

Frequency of intake was reported with the following response options: less than once a month or never; 1–3 times a month; 1–2 times a week; 3–4 times a week; 5–6 times a week; daily, including weekend days. Participants were then asked about the number of servings consumed in a usual day, reporting servings in usual measurements such as units, slices, tablespoons, serving spoons or cups, which were then converted to standardized servings corresponding to 80 g [19, 21].

We estimated the average daily servings of FV for individuals who reported eating FV at least once a month, while intake was set at 0 for those who ate FV less than once a month or never [19]. The number of usual servings was multiplied by the midpoint of frequency categories, i.e., if a participant reported eating 2 servings of vegetables, 3–4 times a week, we multiplied the 2 servings by 3.5. The result was divided by seven if intake happened at least once a week, or by 30 if intake happened more than once a month but less than once a week [19]. Daily fruit intake was added to daily vegetable intake, and the result was multiplied by 80 to estimate daily FV intake in terms of g/day. We classified FV intake as adequate if it reached at least 400 g/day, and as insufficient otherwise [21].

Stages of change were assessed using an algorithm proposed by Kristal et al., and adapted in Brazil by Toral et al. [22, 23]. Individuals were asked about whether they perceived their current FV intake as adequate. When a participant did not perceive their FV intake as adequate, they were asked about their intention to increase it within six months. Those who did not intend to increase FV intake were classified in pre-contemplation, and those with an intention to increase it were asked about their confidence to do so within 30 days. Those who were not confident to increase FV intake within 30 days were classified in contemplation, while those who were confident to do so were classified in preparation. When a participant perceived their FV intake as adequate, they were asked about how long they had maintained their current intake. They were classified in action if they had maintained such intake for under six months, or in maintenance if they had maintained it for six months or more [22, 23]. Due to similarities between some stages of change, they were regrouped as follows: preaction (precontemplation and contemplation stages); preparation (preparation stage); action (action and maintenance stages) [16, 17].

Self-efficacy was assessed using the following statements: “*I can easily buy FV in my neighborhood*”; “*I can buy various FV even when they are expensive*”; “*I can eat the recommended amount of FV*”; “*I have time to prepare FV*”. Response options ranged from “not at all confident” (0 points) to “completely confident” (4 points), and the final score could range from 0 to 16 points [24].

Decisional balance was assessed using the following pros and cons: “*I like the taste of FV*”; “*FV are expensive*”; “*I have time to purchase FV*”; “*I don’t like FV*”; “*Preparing FV is easy*”; “*I don’t have time to eat FV*”; “*Eating FV is good for my body and reduces my disease risk*”; “*I would eat more FV if my family and friends did the same*”. Response options ranged from “completely disagree” (0 points) to “completely agree” (4 points), and the final scores for pros and for cons could range from 0 to 16 points each [25].

Data on FV intake at 48 months were collected using computer-assisted telephone interviews, which have shown substantial agreement compared to face-to-face data collection for estimating fruit intake, and moderate agreement for estimating vegetable intake [20].

### Outcome variable: maintenance of initial changes in FV intake

FV intake at 48 months was compared to FV intake at 12 months to identify whether changes in FV intake were maintained. Maintenance was identified as follows: [0] no maintenance, if FV intake at 48 months was lower than at 12 months; [1] maintenance, if FV intake at 48 months was the same as or higher than at 12 months.

### Data analysis

We performed multiple imputation of missing data on FV intake at 12 and 48 months considering the missing at random mechanism, using sex, age, and schooling as predictors. We employed linear regressions to impute missing data on FV servings, and ordinal logistic regressions to impute data on the frequency of FV intake. Mean values from ten imputed datasets were used in our analyses.

Stata version 14.2 was used for analyses, and significance was set at 0.05. We described participants’ characteristics for the complete sample and according to the maintenance of initial changes in FV intake, using means and 95% confidence intervals (CI) for continuous variables, and proportions and 95% CI for categorical variables. Cross-sectional differences between maintainers and non-maintainers were assessed using bivariate logistic regressions.

FV intake over time was described according to maintenance using line graphs of means and 95% CI. Cross-sectional differences in FV intake between maintainers and non-maintainers were assessed using simple t tests. Changes in FV intake from 12 to 48 months within groups were assessed using paired t tests.

Factors associated with the maintenance of increased FV intake over time were assessed using bivariate and multivariate logistic regression. The following variables were tested with bivariate models: sex; age; schooling; income; hypertension; diabetes mellitus; BMI; attempts to lose weight; receipt of nutrition counseling; time since joining HAP; FV intake; baseline self-efficacy; self-efficacy at 12 months; baseline decisional balance pros and cons; and baseline stages of change for FV intake.

Variables were included in the multivariate model using a backward procedure if bivariate analyses returned a *p*-value < 0.20 or due to theoretical reasons, and maintained when *p* < 0.05. Model 1 included demographics; model 2 included demographics and health-related variables; and model 3 included demographics, health, FV intake, and eating behavior variables. All models were adjusted for sex (binary) and income (continuous). Collinearity was tested with correlation coefficients, and in case of strong correlations (r ≥ 0.35), the variable with fewer missing observations remained in the model.

## Results

Our sample comprised 2232 health promotion service participants, of which 88.4% were women, with an average age of 56.8 years, under eight years of schooling, and with an average income of R$ 887.12. Over half of participants had self-reported hypertension (52.9%), and most reported recent nutrition counseling (59.2%) and attempts to lose weight (60.9%). Average baseline scores were 8.7 for self-efficacy, 12.3 for decisional balance pros, and 6.1 for decisional balance cons, and around 45.5% of individuals were in action stages (data not shown).

**Fig. 2:**
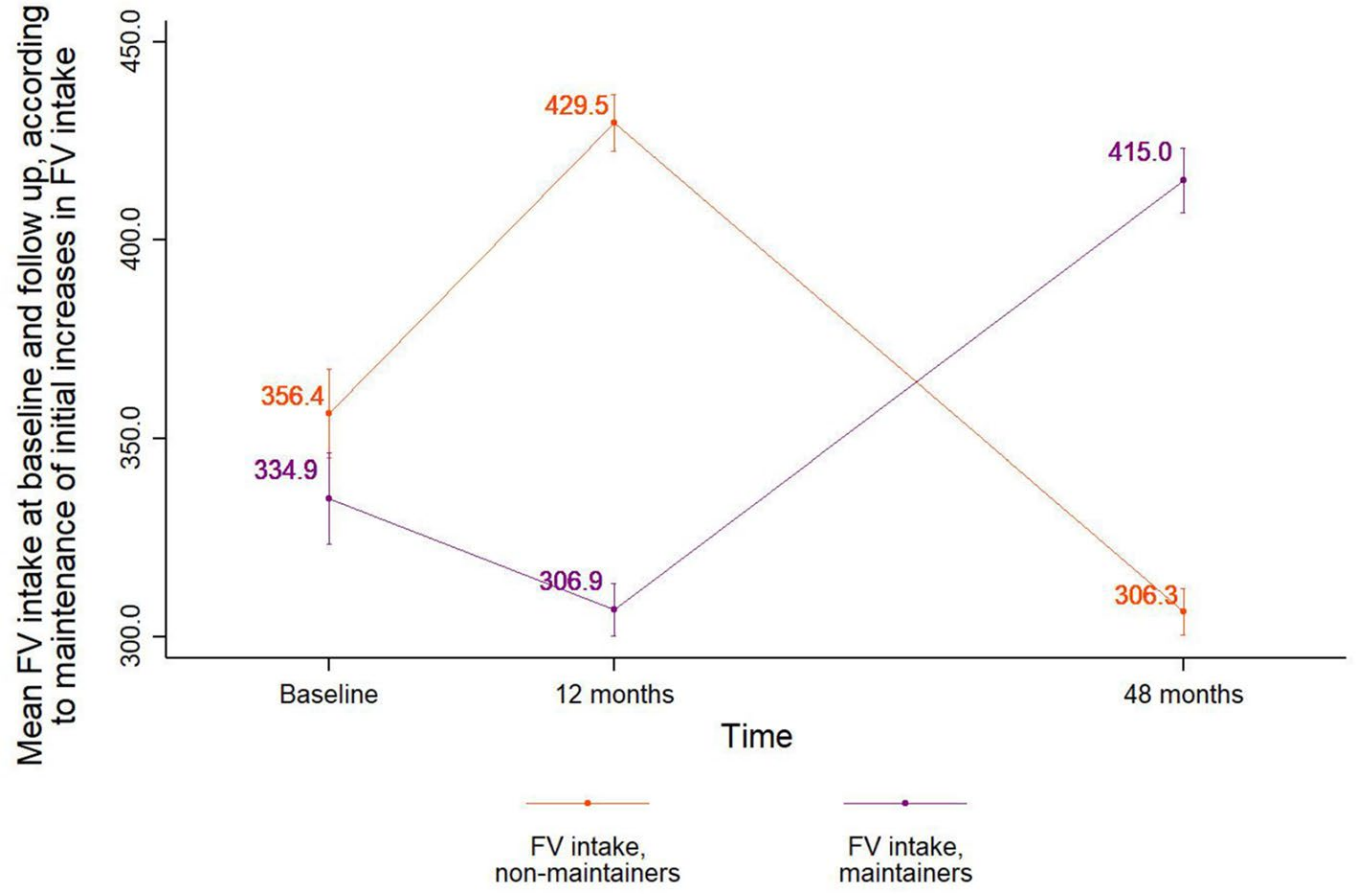
Mean FV intake over time according to maintenance of increased FV intake

Changes in self-efficacy from baseline to 12 months were negative. Average self-efficacy at baseline was associated with maintenance, while self-efficacy scores at 12 months or changes in self-efficacy were not. We identified that 46.6% of participants maintained initial changes in FV intake at 48 months. Maintainers were older (57 vs. 56 years), had lower schooling (7.2 vs. 7.5 years), had been participating in PAS for longer (20.5% vs. 17.4% had been in PAS for 36 months and over), had lower baseline FV intake (334.9 vs. 356.4 g/day), self-efficacy scores (8.5 vs. 8.8), and prevalences of being in preparation (36.8% vs. 37.0%) or action (44.0% vs. 46.7%) groups (Table 1; Fig. 2).

**Table 1:** Characteristics of study participants according to the maintenance of changes in FV intake

Variables	Maintenance of changes in FV intake (*n* = 1.039, 46.6%)		No maintenance of changes in FV intake (*n* = 1.193, 53.4%)		*p* value
	**n**	**(% or mean (95% CI))**	**n**	**(% or mean (95% CI))**	
Sex (%)					0.43
Female	915	88.1% (85.9; 90.0)	1058	88.7% (86.7; 90.4)	
Male	124	11.9% (10.0; 14.1)	135	11.3% (9.6; 13.3)	
Age (years, mean)	1039	57.3 (56.6; 58.0)	1193	56.3 (55.6; 56.9)	** < 0.001**
Schooling (years, mean)	1039	7.2 (7.0; 7.5)	1193	7.5 (7.3; 7.8)	**0.003**
Monthly per capita income (Brazilian Real, mean)	938	870.59 (822.78; 918.41)	1084	901.43 (854.49; 948.38)	0.12
Self-reported hypertension (%)	562	54.1% (51.0; 57.2)	619	51.9% (49.0; 54.8)	0.07
Self-reported diabetes (%)	172	16.6% (14.3; 19.0)	213	17.9% (15.7; 20.2)	0.16
Baseline BMI (kg/m^2^, mean)	998	27.8 (27.5; 28.1)	1140	28.0 (27.7; 28.2)	0.16
Trying to lose weight (%)	630	60.7% (57.6; 63.7)	729	61.1% (58.3; 63.9)	0.73
Received nutrition counseling (%)	606	58.4% (55.4; 61.5)	715	59.9% (57.1; 62.7)	0.22
Time since joining PAS (%)					
0–11 months	402	38.7% (35.7; 41.7)	489	41.0% (38.2; 43.8)	Reference
12–23 months	272	26.2% (23.5; 29.0)	320	26.8% (24.3; 29.4)	0.59
24–35 months	152	14.6% (12.5; 16.9)	177	14.8% (12.9; 17.0)	0.56
≥ 36 months	213	20.5% (18.1; 23.1)	207	17.4% (15.2; 19.6)	**0.001**
Baseline self-efficacy (mean)	1039	8.5 (8.3; 8.7)	1193	8.8 (8.6; 9.0)	**0.001**
Self-efficacy at 12 months (mean)	1039	6.1 (5.8; 6.4)	1193	6.2 (5.9; 6.5)	0.49
Change in self-efficacy from baseline to 12 months	1039	-2.4 (-2.8; -2.1)	1193	-2.6 (-2.9; -2.3)	0.15
Baseline decisional balance—pros (mean)	1039	12.2 (12.1; 12.4)	1193	12.4 (12.3; 12.6)	0.08
Baseline decisional balance—cons (mean)	1039	6.2 (6.0; 6.4)	1193	6.1 (5.9; 6.2)	0.22
Baseline stage of change (%)					
Preaction	199	19.2 (16.9; 21.7)	194	16.3 (14.3; 18.5)	Reference
Preparation	382	36.8 (33.9; 39.8)	442	37.0 (34.4; 39.8)	**0.02**
Action	458	44.0 (41.1; 47.1)	557	46.7 (43.9; 49.5)	**0.001**

Note: 95% CI = 95% confidence intervals. *p*-value from bivariate logistic regression models. Bold denotes significant differences between groups

Maintainers had a lower FV intake at baseline (334.9 g/day) compared to non-maintainers (356.4 g/day, simple t test, *p* < 0.001). They also had a lower intake at 12 months (306.9 g/day) compared to non-maintainers (429.5 g/day, simple t test, *p* < 0.001). However, maintainers increased FV intake to 415.0 g/day at 48 months (paired t test, *p* < 0.001). Meanwhile, non-maintainers decreased their FV intake over time, reaching 306.3 g/day at 48 months (paired t test, *p* < 0.001) (Fig. 2).

Multivariate models revealed that higher age and being in the service for 36 months and over were associated with 1% and 30% higher odds of maintaining changes in FV intake at 48 months. On the other hand, a higher FV intake at baseline and higher self-efficacy scores were associated with 1% and 2% lower odds of maintenance at 48 months (Table 2).

**Table 2:** Factors associated with maintenance of changes in FV intake

Variables	Maintenance of initial increases in FV intake at 48 months (*n* = 1.039)					
	**Model 1**		**Model 2**		**Model 3**	
	**OR (95%CI)**	***p*value**	**OR (95%CI)**	***p*value**	**OR (95%CI)**	***p*value**
Age	**1.01 (1.01; 1.01)**	** < 0.001**	**1.01 (1.01; 1.01)**	**1**	**1.01 (1.01; 1.01)**	** < 0.001**
Time since joining PAS						
0–11 months	NA	NA	Reference		Reference	
12–23 months	NA	NA	0.98 (0.87; 1.12)	0.81	1.00 (0.88; 1.14)	0.96
24–35 months	NA	NA	1.05 (0.90; 1.23)	0.51	1.07 (0.92; 1.25)	0.38
≥ 36 months	NA	NA	**1.25 (1.09; 1.45)**	**0.002**	**1.30 (1.13; 1.50)**	**0.001**
Baseline FV intake	NA	NA	NA	NA	**0.99 (0.99; 0.99)**	** < 0.001**
Baseline self-efficacy	NA	NA	NA	NA	**0.98 (0.96; 0.99)**	**0.011**

Note: 95% CI = 95% confidence intervals. NA = variable not included in the model. *p*-value from bivariate logistic regression models. Bold denotes significant differences between groups. All models adjusted for sex and income

Source: the authors

## Discussion

In a representative sample of Brazilian health promotion service users, around half of participants maintained their changes in FV intake at 48 months. Long-term maintenance was associated with higher age, a longer time since joining PAS, and a lower FV intake and self-efficacy score at baseline.

The strongest association was found between time since joining PAS and maintenance. Long participation in a PHC-based health promotion service might promote individual empowerment to deal with lapses and relapses, which have been frequently identified in follow-up after nutrition interventions aimed at increasing FV intake. Lapse is a temporary return to a previous behavior, after behavior change is initiated, while relapse is a prolonged set of lapses which can lead to a complete return to previous behavior [8, 11].

Individuals who had been in the service for a longer time might have built a stronger rapport with service peers and PHC professionals. Thus, they might feel more confident to seek support when faced with challenges. These individuals might have had adequate physical and external resources to overcome decreases in FV intake and return to an increased FV intake, and PHC professionals might be well equipped to provide comprehensive and longitudinal support for these individuals. Furthermore, adherence to a physical activity routine in a health promotion service setting might have reinforced the adoption and maintenance of another healthy behavior, i.e., increased FV intake, demonstrating the possibility of a transfer effect. This result is similar to the results identified by Fleig et al., in which participants in a physical activity intervention were more likely to also improve their FV intake compared to the control group [26]. Therefore, PAS units can promote the coexistence of desirable behaviors and consistent behavior performance for individuals who increased their FV intake [11, 27, 28].

Age at baseline was positively associated with maintenance, which means it was easier for older individuals to sustain their initial changes in FV intake over time. Previous studies have identified an association between generational membership and FV intake: individuals from the traditionalist generation, born in or before 1945, were more likely to have regular and adequate FV intake when compared to younger groups, especially those from generations X and Y (born from 1965 onwards) [29]. In a qualitative investigation, other researchers identified many facilitators of FV intake that might be associated with age, such as rural origins, valuing a traditional Brazilian eating pattern, having better cooking skills, and being concerned about non-communicable diseases (NCDs) [30, 31].

Higher age has been associated with a trajectory of resilience in maintaining changes in FV intake. Resilience was conceptualized to mean that individuals exposed to lapses and relapses were able to successfully return to adequate FV intake [32]. Furthermore, older individuals in urban areas might spend less time in traffic than their younger peers, and therefore have more time available for routinely purchasing, preparing, and eating FV [33]. An additional factor that can explain the association of higher age with maintenance of FV intake is grit, conceptualized as a personality trait of perseverant and goal-oriented individuals. It has been reported that grit tends to become higher with age, and to facilitate behavior change and its maintenance, which can be favorable for older adults [34].

Older individuals being concerned about NCDs could also reflect anticipated regret, which promotes execution and automaticity of varied healthy behaviors [35]. Additionally, valuing traditional eating habits might be related to intrinsic reward, which means that an individual understands the experience of a given behavior to be rewarding in and of itself. Intrinsic reward does not depend on external incentives, and has been described as a moderator of healthy eating habits formation [35, 36].

Intrinsic reward should be further explored as an intervention target when promoting FV intake. As previously reported, baseline intrinsic reward for eating FV can have positive effects on the strength of FV intake habits after a short follow-up period. It has also been reported that higher intrinsic reward at two weeks amplified the effects of baseline intrinsic reward on habit formation [36]. Since self-chosen behavioral goals are probably more likely to increase intrinsic reward, interventions targeting this mediator should be based on autonomy-promoting theories and techniques [12, 35, 36].

Baseline FV intake was negatively associated with maintenance, which means individuals with a lower intake not only increased their intake at 12 months, but also sustained it over time. This result is in accordance with what was found by Mendonça et al., who identified that the intervention was more effective for individuals in the lowest quartile of baseline FV intake [14]. It also highlights an equity component of PAS, in which the most disadvantaged individuals presented more favorable behavior changes over time [37, 38].

Baseline self-efficacy was also negatively associated with maintenance, which means the least confident individuals were able to overcome this lack of confidence, increasing their FV intake at 12 months and sustaining it over time. The potential association of baseline self-efficacy and maintenance of behavior change is understudied and controversial, with some studies reporting that self-efficacy might facilitate habit formation, while others have not identified such a relationship [35]. It is noteworthy that individual self-efficacy is subject to substantial change from day to day, which can explain the different ways that baseline self-efficacy and evolving self-efficacy can be associated with behavior change [35]. Another explanation for this association might be an issue of “excessive confidence”, in which individuals with higher levels of baseline self-efficacy for behavior change might not be well-prepared to face unpredicted barriers for behavior change maintenance over time [35]. Other psychosocial constructs, such as grit, might also be associated with maintenance, but were not measured in our study [34]. Furthermore, food environments around PAS units have been shown to be unfavorable for healthy eating, which might have posed an unexpected barrier for individuals with initially higher self-efficacy [39].

The large sample size, long-term follow-up, and the mechanisms employed for data quality assurance and control should be highlighted as strengths of this study. On the other hand, FV intake was self-reported, and therefore subject to a social desirability bias. However, the brief instrument for estimating FV intake was validated with our population, for use in both face-to-face and telephone-based interviews [19, 20]. There was also a relevant percentage of missing outcome data during follow-up, which is expected in longitudinal studies. To address this limitation, we analyzed our data according to the intention-to-treat approach, using multiple imputation of missing outcome data.

## Conclusions

We conclude that older individuals, those who have been enrolled in a health promotion service for longer, and who had lower FV intake or self-efficacy at baseline were more likely to maintain increases in FV intake over time. Health promotion services within PHC contexts and research groups are encouraged to tailor their interventions to participants’ characteristics. It is necessary to pay special attention to those who might face more barriers for maintaining initial increases in FV intake, such as younger individuals, those with a shorter time of participation, and those who present higher self-efficacy scores. Skill development, problem solving, and relapse prevention can be useful behavior change techniques to emphasize during intervention with these subgroups.

## Data Availability

The datasets used and/or analyzed during the current study are available from the corresponding author on reasonable request.

## Abbreviations

FV: Fruit and vegetables
PHC: Primary Health Care
TTM: Transtheoretical Model
RCCT: Randomized controlled community trial
PAS: Programa Academia da Saúde
HAP: Health Academy Program
CI: Confidence intervals
BMI: Body mass index
NCDs: Non-communicable diseases
